# Before the Lords' Committee

**Published:** 1890-06-21

**Authors:** 


### Before the Lords' Committee.

(By Our Special Commissioner.)

### IV.—MR. WILLIAM BOUSFIELD AND DR. W. B. CLARK.

### Critical and Suggestive.

Mr. Bousfield, who was examined on the 15th of May, gave his evidence with great fairness and some fullness of knowledge. He is probably the most accurate witness who has yet been examined before the committee. His evidence had one great defect like that of some of the witnesses who preceded him—much of what he said was ancient history. Thus: "I am speaking now of an inquiry made at least ten years ago, and I cannot tell whether things are the same now." May we once more, without offence, suggest that it is due to the Committee and due to the credibility and self-respect of every witness to prepare himself for examination, so as to bring any information he has to give absolutely up to date?

### Out-Patients and Casualty Cases.

It appears that in 1876 a skilled officer was appointed to sit in the out-patient department of King's College Hospital to determine how far the applicants were fit objects of relief. Doubtful cases were sent to the Charity Organisation Society. These appear to have been very few owing, no doubt, to the fact that it caused several day's delay. The rapidity with which patients were seen at King's College, according to Mr. Bousfield, exceeds that of every other hospital, for he stated that three patients in certain cases were seen in a minute, though a considerable time was given to certain interesting cases. An examination proved that many of the applicants practically required no medicine at all. For such cases "a stock bottle, composed of materials which were supposed to be medicine, was served out in order to satisfy the patients when they went away." Mr. Bousfield had no doubt that there were cases of persons who have nothing particular to do, and who come to the out-patient department to have a chat with the possibility of a drink from this stock bottle afterwards. He knew that both out-patients and casualty cases were treated by students. If the out-patient departments were abolished the cases would find their way to provident dispensaries and Poor Law Infirmaries. Mr. Bousfield expressed the view that it was impracticable to open the out-patient department in the evening, and afterwards gave a description of the system adopted by the Metropolitan Provident Medical Association, which has already been referred to at length in these columns, and need not therefore be repeated.

### Does Enquiry Keep Down the Numbers?

At King's College it would appear that, for some years the effect of the inquiry was to diminish the numbers. Thus, in 1871 there were 33,111 out-patients. In 1877, after the appointment of a skilled officer, the numbers fell to 20,337, and in 1880 they only amounted to 14,069. When the novelty wore off the numbers began to increase again, and last year there were 19,592 out-patients, including casualties. This increase is due to the fact that the poor found out that by going to the casualty department they had immediate treatment at any time without any enquiry, the consequence being that the numbers of that department have very much increased and are very much increasing, the "stock bottle" notwithstanding. At the present time the out-patients proper only amount to 8,447, whereas the casualties have risen to 10,439. Casualties are supposed to be accidents and sudden cases of emergency, but these apparently form at the present time only a small proportion of those treated in the casualty department. Mr. Bousfield is quite right in stating that the dress worn by a patient is no indication of his circumstances or position, and he made a point by declaring that patients have a very clear idea in their own minds whether they are fit subjects for free medical relief or not. The working classes readily admit that those earning good wages and who are fairly well off ought not to seek out-patient relief. Such cases, and the great proportion of the casualty cases ought, in Mr. Bousfield's opinion, to be treated at the provident dispensaries, or by medical practitioners amongst the poor, or at Poor Law dispensaries.

### Territorial Areas.

Mr. Bousfield was of opinion that co-operation amongst all the medical charities in a given district was not only desirable but necessary in the interests of the institutions as well as of the patients and subscribers. Thus a large general hospital ought to work in co-operation with the special hospitals and dispensaries (provident and Poor Law), situated in the district which they served. He was opinion that if a comprehensive scheme of the kind was promulgated by the Charity Commissioners or some other responsible body, voluntary hospitals would gradually see their way to falling in with it. It might be possible to remove some of the hospitals. Special hospitals had been formed very much to the detriment of the larger institutions. They were started, said Mr. Bousfield, by doctors only anxious for the promotion of their own reputation, and had thus taken away much-needed money from the general hospitals. Certain metropolitan Poor Law infirmaries, like the Marylebone and Kensington Infirmaries, were admirable hospitals, and provided nurses almost equal to the general hospitals, although their numbers were far less in proportion at the infirmaries themselves. He thought that an infirmary, like that at Kensington, of 700 beds, ought to have a visiting staff of physicians and surgeons like the hospitals, as he considered the present medical staff of two resident medical officers insufficient.

### Casualty Department at Bart.'s.

Dr. Clark, of St. Bartholomew's Hospital, stated that for every out-patient seen at that hospital, there were at least six casualty cases. He defined a casualty case as "anyone who comes to the hospital for the moment," and every case is spoken of as being passed through the casualty department. A "casualty" may become an out-patient or an in-patient. Fifty or sixty patients were seen in an hour with the aid of ten house surgeons and assistant house surgeons, and forty dressers. It is absolutely erroneous, so far as surgical cases go, to say that each patient would only have about a minute. In seven days 619 male and 257 female patients were entered in the casualty book at Bart.'s. Medical practitioners who receive a fee of five shillings a visit very often find that their patients go to the hospital.

### The Residential College at Bart.'s., etc.

Dr. Clark was in favour of the reduction in the number of medical schools, though he thought it would be a mistake to amalgamate all the schools. He could not recall a single instance of a medical student being expelled or rusticated for bad conduct from any hospital, thus confirming what Dr. Steele had said previously. The residential college at St. Bartholomew's had accommodation for from forty to fifty students, and the demand for admission was so great that it was always full. It had existed for thirty-five years, having been instituted by Sir James Paget originally, and being the oldest medical college in London.

